# Translating culture into design practice: a preliminary test of a dual-cultural framework for animation

**DOI:** 10.3389/fpsyg.2026.1882608

**Published:** 2026-10-20

**Authors:** Yuqin Zhang, Junghyun Heo

**Affiliations:** 1 Laboratory Meta Interaction Design, The Graduate School of Techno Design, Kookmin University, Seoul, Republic of Korea; 2 Laboratory Intelligent Interaction Design, School of Art and Design, Sanming University, Sanming, China; 3 Department of AI Design, College of Design, Kookmin University, Seoul, Republic of Korea

**Keywords:** 4P taxonomy, animation design, cross-cultural design, cultural layers, dual-cultural framework, structural equation modeling (SEM)

## Abstract

**Introduction:**

How can abstract cultural concepts be translated into animation design practice? This study addresses this question by proposing a dual-cultural framework that integrates a layered conception of culture with a content-oriented 4P taxonomy.

**Methods:**

Using survey data from 368 animation and related industry professionals recruited through relevant enterprises in mainland China, structural equation modeling (SEM) was applied to examine professionals' perceived relationships between cultural dimensions and five animation design elements.

**Results:**

The structural model showed satisfactory fit (χ^2^/df = 1.165, RMSEA = 0.021, CFI = 0.983). The results indicated that Deep Culture was significantly associated with Narrative (β = 0.326), and Surface Culture was associated with Visual Expression (β= 0.233) and Interaction/Immersion (β = 0.217). The 4P dimensions also showed significant associations with corresponding design dimensions, including Product with Visual Expression, Place with Spatial Design, People with Character Design, and Performance with Narrative and Interaction/Immersion.

**Discussion:**

These findings provide preliminary, perception-level evidence for differentiated relationships between culturally framed constructs and animation design dimensions. Finally, an illustrative analysis of *I Am What I Am* (2021) was used to contextualize how selected relationships may be represented in animation practice.

## 1 Introduction

Appadurai (1996) noted that globalization has contributed to the increasing circulation of culture across national and geographical boundaries. In today's globalized world, animations have become one of the important media through which culture is represented and communicated. As a blend of technology and art, animation enables creators to communicate meaning related to identity, traditional culture, and societal values through visual and narrative forms. Condry (2013) highlighted the distinctive collaborative production processes and media ecology of animation. Within this production context, animated works can communicate not only immediately recognizable visual symbols but also less visible cultural meanings embedded in narratives, characters, and aesthetic forms (Lamarre, 2009; Wells, 2002). However, cultural representation in animation may sometimes emphasize readily recognizable visual elements or stereotypical representations at the expense of more complex cultural meanings (Allison, 2008). Such an emphasis on visual representation can simplify the cultural system underlying animated narratives and limit the communication of culturally situated meanings. Leong and Clark (2003) identified a continuing gap between basic cultural knowledge and its practical application in the design process, highlighting the need for more systematic approaches to translating cultural knowledge into design practice.

For designers, translating regional culture into tangible design elements is one challenge (Lin, 2007), while integrating such cultural meanings into a coherent digital narrative presents another. Faced with the increasing complexity of contemporary digital media production, existing approaches may not fully account for the multidimensional process through which cultural knowledge is related to design practice. Recent studies have therefore emphasized the need to move beyond treating culture primarily as a visual or symbolic “texture” and to consider more systematic approaches to cultural representation in digital design (Jie et al., 2024; Roth, 2021). Based on this, the research objective of this paper is to construct and preliminarily test a dual cultural analysis framework to bridge the gap between culture and design practice. Our approach integrates complementary analytical perspectives: cultural layers and cultural content categories. The first perspective concerns cultural depth and is informed by layered conceptions of culture that distinguish observable cultural manifestations from less visible values, norms, and underlying assumptions (Hall, 1976; Schein, 2010). Drawing on these conceptions, the present study operationalizes cultural depth as Deep Culture, Intermediate Culture, and Surface Culture in the context of animation design.

The second perspective concerns the types of cultural content through which cultural meanings may be represented. For the purposes of this study, these content categories are organized into a 4P taxonomy comprising Product, Place, People, and Performance. Rather than treating the 4P taxonomy as an established anthropological model, the present study uses these four categories as a literature-informed analytical organization of cultural content. Product concerns tangible cultural artifacts and designed objects; Place concerns culturally meaningful geographical and spatial contexts; People refers to social identities, roles, and culturally patterned behavior; and Performance encompasses embodied cultural practices such as rituals, festivals, and ceremonies (Lin, 2007; Tuan, 1977; Matsumoto, 2006; Turner, 1982). However, existing research provides limited integration of these two complementary perspectives in the context of animation design. Cultural-layer approaches primarily address the depth or level of cultural meaning, whereas content-oriented categories address the forms through which cultural meanings are manifested. Integrating these perspectives therefore allows cultural depth and cultural content to be considered within a common analytical framework.

Furthermore, although qualitative research has provided valuable insights into cultural representation and design practice, comparatively limited quantitative research has examined the relationships between differentiated cultural dimensions and specific animation design dimensions within an integrated framework. To examine these hypothesized relationships, we apply quantitative analysis and structural equation modeling (SEM) to analyze the multidimensional associations between the culturally framed constructs and animation design dimensions. SEM provides an appropriate analytical approach for simultaneously evaluating relationships among multiple latent constructs (Anderson and Gerbing, 1988).

This focus is also relevant to media psychology. Previous research has examined psychological processes associated with mediated narratives, including narrative transportation (Green and Brock, 2000), narrative engagement (Busselle and Bilandzic, 2009), and character identification (Cohen, 2001).

In a recent experimental study, Lu et al. (2024) examined children's responses to different presentations of animated characters and found differences in narrative engagement across character-presentation conditions. In a study of the reception of Disney's 2020 live action film Mulan, Shen and Yu (2025) analyzed Chinese viewers' online comments and found that some viewers questioned the film's fidelity to The Ballad of Mulan and its representation of Chinese history and culture, illustrating the relevance of cultural authenticity to cross-cultural media reception. In an experiment comparing the viewing of a geometric-shape animation on a conventional screen and in virtual reality, Marañes et al. (2024) measured viewers' sense of presence but found no significant differences between the two conditions on the items assessing the feeling of “being there” and “being present.”

These perspectives provide a broader psychological context for the Narrative, Character Design, and Interaction/Immersion dimensions considered in this study. However, the present research examines animation and related industry professionals' perceptions of design relationships rather than directly measuring audience transportation, identification, presence, or cultural authenticity.

## 2 Literature review

### 2.1 Cultural layers in design

To examine how culture can be translated into media and design practices, layered conceptions of culture provide a useful analytical perspective. Culture involves both observable expressions and less visible meanings. For example, Geertz (1973) interpreted culture as a system of meanings expressed through symbolic forms, while Hall (1976) emphasized the implicit and contextual dimensions of culture and communication. These perspectives provide a broader basis for understanding culture beyond its immediately visible manifestations. Related layered conceptions can also be found in Schein's levels of culture (1985) and Trompenaars and Hampden-Turner's onion model (1997). Drawing on these layered perspectives, the present study organizes cultural depth into three analytical levels: Deep, Intermediate, and Surface Culture. Deep Culture includes philosophies and belief systems underlying cultural values. Intermediate Culture concerns daily routines, social structures, and behavioral norms, while surface culture includes observable expressions such as visible signs, dress, and visual symbols (Schein, 1985; Trompenaars and Hampden-Turner, 1997). In the context of cross-cultural product design, Lin (2007) similarly described three cultural levels: “outer layer (material culture like symbol), middle layer (behavioral culture like ritual, custom, practical norm) and inner layer (spiritual culture like philosophy concept and value)” (p. 46).

Lin (2007) emphasized the importance of translating cultural characteristics into forms applicable to creative design practice. Barthes (1977) suggested that surface design elements should not be regarded as mere decorative features, but rather as semiotic markers, acting as visual codes that can convey broader cultural meanings. This means that cultural knowledge can be translated into design thinking and practice to achieve structural coherence (Leong and Clark, 2003). Norman's (2004) famous three-element emotional design model also provides a related perspective on different levels of design experience. Norman distinguishes visceral, behavioral, and reflective levels of design. At the same time, when discussing cultural behavior, Matsumoto pointed out that subtle non-verbal cues, such as body language and facial expressions, often carry important meanings within specific cultural contexts (2006). Without a clear approach to cultural translation in cross-cultural design, connections between the cultural meanings and surface-level aesthetic expression may remain underdeveloped.

### 2.2 The role of the 4P taxonomy in cultural translation

In this study, the 4P taxonomy is used to organize cultural content into four analytical categories: Product, Place, People, and Performance. Rather than treating the 4P taxonomy as an established anthropological model, these categories provide a content-oriented perspective for examining how cultural elements can be represented in animation design. Holt (2004) argued that cultural expression can involve giving concrete form to broader social meanings and cultural narratives. As mentioned by Lin (2007), translating cultural characteristics into contemporary design involves identifying both tangible and intangible cultural elements.

The 4P taxonomy used in this study should also be distinguished from McCarthy's (1960) marketing framework of Product, Price, Place, and Promotion. In the present framework, Product, Place, People, and Performance are used as analytical categories of cultural content rather than as a marketing strategy. Design anthropology also provides a broader perspective on the relationship between cultural knowledge and design practice (Gunn et al., 2013). The following sections describe each dimension in relation to cultural content and design.

(a) **P**roduct reflects cultural memory. Drawing on Appadurai's (1986) perspective on the social life of things, cultural artifacts can be understood not only as material objects but also in relation to the social and cultural meanings they carry.
(b) **P**lace reflects the characteristics of space and the built environment. Drawing on Tuan's (1977) distinction between space and Place, Place in the present framework refers to spatial and environmental settings that carry cultural meanings.
(c) **P**eople refers to social identities and patterns of social and embodied behavior. The concept of “habitus” proposed by Bourdieu (1977) provides a relevant perspective for understanding how social structures are expressed through individual dispositions, social practices and embodied behaviors.
(d) **P**erformance consists of dynamic rituals, cultural practices, narrative events, and sequences. Based on Turner's (1982) anthropological performance theory, performance provides a context in which cultural values and social meanings can be expressed and reaffirmed.

### 2.3 Animation as a medium for constructing cultural narratives

Animation design is not only a canvas for stylistic experimentation, but also a vehicle embedded in cultural narratives. Wells (2002) highlighted the expressive potential of the animation medium in representing cultural meanings through moving images. Japanese animation provides a prominent example of this potential. As Napier (2006) argues, unique geographical spaces and narrative mechanisms can play important roles in cultural representation. These elements can bring regional cultural meanings into animated narratives and contribute to their communication across different cultural contexts.

If animation focuses primarily on visual spectacle, its potential as a vehicle for communicating cultural meanings may be overlooked. Allison (2008) provides a relevant perspective on the representation of tradition and cultural meanings in Japanese popular media. Through the use of visual metaphor and narrative expression, less visible cultural meanings can be represented through media forms. At the same time, traditional narrative forms are being reshaped through digitization. Holliday (2018) and Ryan (2015) discussed the expanding possibilities of digital and immersive media beyond conventional forms of narrative expression. Translating local stories into digital forms may also create challenges in preserving their cultural meanings (Zhen et al., 2024). These challenges highlight the need for a design framework that considers cultural meaning alongside the technical and expressive possibilities of digital animation.

### 2.4 Research gap and the construction of the dual-cultural framework

Classic models such as Hofstede (2001), Schein (2010), and Trompenaars and Hampden-Turner (2011) provide important theoretical foundations for understanding cultural differences and cultural structure. However, their relatively abstract concepts can be difficult to translate directly into specific animation design decisions. This is not a flaw of these theories; rather, it reflects a gap between cultural analysis and design application. At the same time, content-oriented categories such as the 4P taxonomy can help identify observable cultural elements but provide less guidance regarding differences in cultural depth. A framework connecting these two analytical perspectives is therefore needed. To address this particular gap, we propose a dual-cultural framework that integrates Deep, Intermediate, and Surface Culture as levels of cultural depth with the 4P taxonomy (Product, Place, People, Performance) as categories of cultural content. These two complementary perspectives are then related to five animation design dimensions—Narrative, Character Design, Visual Expression, Spatial Design, and Interaction/Immersion. This provides a structured analytical basis for examining the relationships between cultural factors and animation design dimensions. Finally, structural equation modeling (SEM) (Anderson and Gerbing, 1988) is used to examine the hypothesized relationships within the proposed framework.

## 3 Research design and structural equation modeling

### 3.1 Conceptual framework

Translating abstract cultural knowledge into something tangible that can be applied in animation design remains a significant challenge (Condry, 2013; Glăveanu, 2011; Leong and Clark, 2003). To address this challenge, this paper puts forward a dual-cultural framework. Instead of being just a simple description of localized cultural heritage (Lin, 2007; Zhen et al., 2024), it provides an analytical matrix. The framework considers cultural depth in terms of the three levels—Deep, Intermediate, and Surface culture—drawing on layered conceptions of culture (Hofstede, 2001; Schein, 2010), together with a content-oriented 4P taxonomy comprising Product, Place, People, and Performance. Based on current theories regarding new media and digital storytelling (Manovich, 2001; Ryan, 2015), the matrix is then mapped structurally onto five factors that relate to design aspects of animation: Narrative, Character Design, Spatial Design, Visual Expression and Interaction/Immersion. In the present study, these factors are treated as design-related constructs rated by animation and related industry professionals rather than as direct measures of audience psychological responses. The proposed relationships were then examined using SEM (Anderson and Gerbing, 1988).

### 3.2 Variables and hypotheses

#### 3.2.1 Independent variables

To operationalize the framework, cultural layers and the 4P taxonomy are treated as independent variables. Within the SEM context, they are modeled as latent constructs representing the cultural factors hypothesized to be associated with animation design outcomes. As outlined in Table 1, these theoretical concepts are operationalized as corresponding constructs within the proposed framework.

**Table 1:** Independent variables (latent constructs).

Dimension	Construct	Definition	Example
Cultural layers	Deep Culture (D)	Core values, belief systems, and cosmological views	Philosophical principles guiding the overarching narrative
Cultural layers	Intermediate Culture (I)	Social rituals, interpersonal norms, and spatial behaviors	Etiquette, communal interactions
Cultural layers	Surface Culture (S)	Visual symbols, fashion, language	Costumes, artifacts, linguistic motifs
4P content	Product (P1)	Tangible crafts and physical objects	Traditional props, tools, and iconic items
4P content	Place (P2)	Architecture and natural settings	Temples, landscapes, and distinct ecosystems
4P content	People (P3)	Social identities, community customs, and demographics	Archetypal characters and community behavioral patterns
4P content	Performance (P4)	Dynamic rituals, festivals, and performing arts	Traditional dance, music, ceremonies, and storytelling

#### 3.2.2 Dependent variables

Corresponding to the cultural independent variables in the structural model, five animation design dimensions are treated as dependent variables (endogenous constructs): Narrative, Character Design, Visual Expression, Spatial Design, and Interaction/Immersion. These constructs represent design-related dimensions of animation and are informed by earlier media theories (Napier, 2006; Ryan, 2015; Wells, 2002). Table 2 provides the definitions and theoretical assumptions underlying these dependent variables.

**Table 2:** Dependent variables (latent constructs).

Construct	Definition	Linked cultural dimension
Narrative (A1)	Thematic coherence and cultural depth of storylines	Deep culture, Performance
Character Design (A2)	Representation of cultural identity in characters	Intermediate culture, People
Visual Expression (A3)	Aesthetic fidelity achieved through cultural motifs, colors, and patterns	Surface culture, Product
Spatial Design (A4)	Representation of cultural and geographical norms within environmental settings	Deep Culture, Intermediate Culture, Place
Interaction/ Immersion (A5)	Participatory engagement and rhythm inspired by cultural practices	Surface culture, Performance

#### 3.2.3 Research hypotheses

To examine the above theoretical framework, this section proposes specific hypotheses that connect cultural factors with design outcomes. Based on the theoretical sources of the independent variables, these hypotheses are divided into two groups: H1–H3 examine the hypothesized relationships involving cultural layers (Deep, Intermediate, and Surface); H4 examines the hypothesized relationships involving the 4P taxonomy. The overall hypothesized paths are detailed in Figure 1 and Table 3.

**Figure 1:**
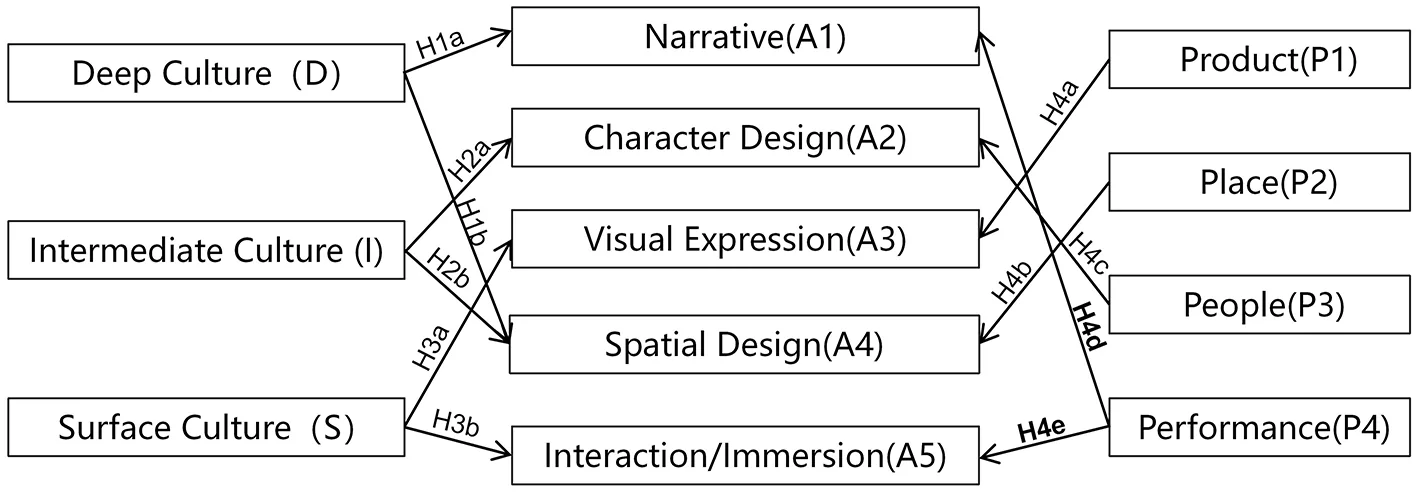
Conceptual framework diagram. It shows how deep, intermediate, and surface culture inform key animation design elements and connect them with the 4P dimensions: product, place, people, and performance.

**Table 3:** Hypotheses.

Code	Hypothesis
H1a	Deep Culture (D) positively associated with Narrative (A1).
H1b	Deep Culture (D) positively associated with Spatial Design (A4).
H2a	Intermediate Culture (I) positively associated with Character Design (A2).
H2b	Intermediate Culture (I) positively associated with Spatial Design (A4).
H3a	Surface Culture (S) positively associated with Visual Expression (A3).
H3b	Surface Culture (S) positively associated with Interaction/Immersion (A5).
H4a	Product (P1) positively associated with Visual Expression (A3).
H4b	Place (P2) positively associated with Spatial Design (A4).
H4c	People (P3) positively associated with Character Design (A2).
H4d	Performance (P4) positively associated with Narrative (A1).
H4e	Performance (P4) positively associated with Interaction/Immersion (A5).

### 3.3 Measurement development

This study draws on theoretical literature from fields including cultural design and media psychology to operationalize the latent structures as summarized in Table 4. These sources informed the operationalization of creativity-related evaluation, place-based meaning, and narrative structure in the measurement framework (Amabile, 2018; Hennessey et al., 2011; Kavaratzis and Ashworth, 2005; Moilanen and Rainisto, 2009; Todorov, 1968). Each construct consists of three observed items, all rated using a 5-point Likert scale (1 = strongly disagree, 5 = strongly agree). To ensure construct validity, the measurement items were contextualized for animation-design scenarios while retaining the core conceptual meanings of the corresponding constructs. The content validity of the preliminary questionnaire was assessed by three experts in animation design and cultural studies. Based on their qualitative feedback, minor modifications were made to the wording and the order of items to reduce ambiguity. Because the questionnaire was originally developed in Chinese, an English version was subsequently prepared for reporting purposes, with the wording checked to maintain consistency with the meaning of the original Chinese items. For a detailed psychometric assessment of the final measurement indicators (including Cronbach's Alpha, CFA, and composite reliability), see Section 3.5.

**Table 4:** Construct operationalization and theoretical basis.

Construct	Example item	References
Deep Culture	“The animation reflects philosophical or cosmological values.”	Hofstede, 2001; Schein, 2010
Intermediate Culture	“Character interactions follow recognizable social rituals.”	Schein, 2010; Matsumoto, 2006
Surface Culture	“Colors and patterns reflect regional cultural identity.”	Wells, 2002; Schein, 2010
Product	“Props and objects embody tangible cultural meaning.”	Lin, 2007; Ho et al., 2009
Place	“Environmental settings reflect culturally meaningful places and geographical characteristics.”	Tuan, 1977; Lin, 2007
People	“Character behavior reflects traditional community norms.”	Matsumoto, 2006
Performance	“The rhythm of action reflects ritual or performative practices.”	Turner, 1982
Narrative	“The storyline conveys cultural themes and connotations.”	Ryan, 2015; Roth, 2021
Character Design	“Character design reflects culturally recognizable identities and social characteristics.”	Wells, 2002; Matsumoto, 2006
Visual Expression	“The design themes are consistent with cultural aesthetics.”	Lin, 2007; Zhen et al., 2024
Spatial Design	Spatial settings reflect culturally and geographically meaningful characteristics.	Tuan, 1977; Lin, 2007
Interaction/ Immersion	“This interactive rhythm design is inspired by cultural rituals.”	Ryan, 2015; Yoshinaga, 2020

### 3.4 Sample and data collection

A purposive sampling survey was conducted online on April 21, 2025, with the questionnaire distributed through WeChat to professionals recruited through relevant enterprises in mainland China. The relevant enterprise WeChat groups had an estimated combined membership of approximately 421; however, the number of individuals who actually received or viewed the invitation could not be verified. A total of 368 respondents completed the questionnaire. All 368 respondents reported that they were engaged in animation- or culture-related fields, as indicated by the background question included in the questionnaire. Before responding to the measurement items, participants were instructed to base their judgments on their viewing experience of culturally themed animation. The questionnaire provided ***Spirited Away, Big*** ***Fish & Begonia***, **and** ***Ne Zha*** (*Ne Zha: Birth of the Demon Child*) as reference examples, while respondents could also draw on other culturally themed animated works familiar to them. The final dataset comprised 368 usable questionnaires (*N* = 368). The analytical dataset contained no missing values, and no observations were excluded through outlier screening. Table 5 summarizes the demographic characteristics of the sample. Among the respondents, 59.5% were male, and 71.2% were aged between 25 and 44 years.

**Table 5:** Basic information survey results.

Characteristic	Category	Frequency	Percentage (%)
1. Gender	Male	219	59.5
	Female	149	40.5
2. Age group	18–24	73	19.8
	25–34	97	26.4
	35–44	165	44.8
	45+	33	9.0
3. Professional background	Yes (culture—related fields)	368	100

The study involved a voluntary, anonymous, minimal-risk online questionnaire and did not collect personally identifiable information. Under the applicable institutional requirements of Kookmin University, a formal Institutional Review Board (IRB) application was not required. Before beginning the questionnaire, participants were informed of the academic purpose of the study, the voluntary and anonymous nature of participation, and their right to withdraw before submission. Participants indicated their informed consent electronically before proceeding to the questionnaire items. The questionnaire did not collect respondents' specific job titles or years of professional experience.

### 3.5 Measurement model analysis

#### 3.5.1 Reliability analysis

Cronbach's alpha was used to assess the internal consistency of each construct. As reported in Table 6, Cronbach's α values for the 12 constructs range from 0.762 to 0.882, exceeding the commonly accepted threshold of 0.70. Visual Expression showed the highest internal consistency (α = 0.882), whereas Spatial Design had the lowest coefficient (α = 0.762), which nevertheless remained above the recommended threshold. Overall, the results indicate satisfactory internal consistency across all constructs and support their use in the subsequent measurement-model analysis.

**Table 6:** Internal consistency reliability (Cronbach's α).

Construct	Cronbach's α	Number of items
Deep Culture (D)	0.809	3
Intermediate Culture (I)	0.821	3
Surface Culture (S)	0.853	3
Product (P1)	0.810	3
Place (P2)	0.806	3
People (P3)	0.836	3
Performance (P4)	0.826	3
Narrative (A1)	0.794	3
Character Design (A2)	0.818	3
Visual Expression (A3)	0.882	3
Spatial Design (A4)	0.762	3
Interaction/Immersion (A5)	0.815	3

#### 3.5.2 Analysis of validity

Construct validity was evaluated through a sequential procedure combining an exploratory assessment of the item structure and confirmatory factor analysis (CFA). Content validity was established by the expert-panel review described above. The empirical construct structure of the measurement items was first examined using principal component analysis (PCA), followed by CFA to evaluate the hypothesized 12-construct measurement model. Convergent and discriminant validity were subsequently assessed using standardized factor loadings, average variance extracted (AVE), composite reliability (CR), the Fornell–Larcker criterion, and the heterotrait–monotrait ratio of correlations (HTMT). PCA and CFA were conducted using the same sample of 368 respondents; consequently, the CFA findings are interpreted as a preliminary assessment of the proposed measurement model rather than independent validation.

##### 3.5.2.1 Exploratory assessment of the measurement structure

Data suitability was statistically assessed before performing PCA. The Kaiser–Meyer–Olkin (KMO) measure of sampling adequacy yielded a value of 0.810 (Table 7). Simultaneously, Bartlett's Test of Sphericity exhibited high statistical significance (Approximate χ^2^ = 5850.946, df = 630, *p* < 0.001), rejecting the null hypothesis of an identity correlation matrix and indicating sufficient correlation among the variables.

**Table 7:** KMO and Bartlett's test.

Test	Statistic	Value
*KMO measure of sampling adequacy*		0.810
*Bartlett's test of sphericity*	Approximate Chi-Square	5,850.946
	Degrees of Freedom (*df*)	630
	Significance (*p*-value)	< 0.001

After checking data suitability, principal component analysis (PCA) was performed to examine the underlying structure of the item set, using the conventional criterion of retaining components with eigenvalues > 1.0. The analysis retained 12 components in total. The eigenvalue-greater-than-one criterion was used as an initial component-retention rule rather than as conclusive evidence for the proposed 12-construct structure.

Importantly, the resulting structure was consistent with the theoretical framework, corresponding to three cultural layers, the 4P content categories, and the five animation creative dimensions. As shown in Table 8, the 12-component solution explained 74.779% of the total variance. As this value exceeds the commonly used 60% threshold, the solution indicates substantial cumulative variance coverage.

**Table 8:** Results of eigenvalues and cumulative variance explained.

Element	Initial eigenvalue			Rotation sums of squared loadings		
	Total	Variance percentage	Cumulative %	Total	Variance percentage	Accumulate %
1	6.754	18.762	18.762	2.469	6.859	6.859
2	3.089	8.579	27.341	2.282	6.339	13.198
3	2.692	7.478	34.820	2.267	6.297	19.494
4	2.406	6.684	41.503	2.251	6.253	25.748
5	2.248	6.245	47.748	2.246	6.240	31.987
6	1.818	5.051	52.799	2.244	6.234	38.222
7	1.643	4.565	57.365	2.241	6.226	44.448
8	1.531	4.253	61.617	2.218	6.160	50.608
9	1.302	3.616	65.233	2.215	6.154	56.762
10	1.231	3.420	68.653	2.212	6.144	62.905
11	1.163	3.231	71.884	2.190	6.085	68.990
12	1.042	2.895	74.779	2.084	5.789	74.779
13	0.582	1.618	76.397			
14	0.570	1.584	77.981			
15	0.535	1.487	79.468			
16	0.521	1.448	80.916			
17	0.481	1.337	82.253			
18	0.475	1.320	83.573			
19	0.458	1.272	84.845			
20	0.438	1.217	86.062			
21	0.426	1.182	87.244			
22	0.395	1.098	88.342			
23	0.378	1.049	89.392			
24	0.375	1.042	90.434			
25	0.362	1.006	91.440			
26	0.352	0.989	92.429			
27	0.345	0.959	93.387			
28	0.331	0.919	94.307			
29	0.306	0.849	95.156			
30	0.292	0.810	95.966			
31	0.276	0.765	96.731			
32	0.272	0.757	97.488			
33	0.263	0.731	98.219			
34	0.247	0.686	98.905			
35	0.224	0.621	99.526			
36	0.170	0.474	100			

##### 3.5.2.2 Component loadings and construct validity

The exploratory analysis identified 12 distinct components, which were well aligned with the proposed dual-cultural framework. As shown in Table 9, all 36 measurement items loaded strongly on their corresponding components. Specifically, the dominant rotated component loading ranged from 0.750 to 0.913, and the communalities ranged from 0.669 to 0.866, indicating a clear and interpretable measurement structure.

**Table 9:** Rotated component matrix and item communalities.

Item	1	2	3	4	5	6	7	8	9	10	11	12	Communalities
DCU1						0.791							0.727
DCU2						0.838							0.763
DCU3						0.791							0.719
ICU1				0.820									0.729
ICU2				0.825									0.743
ICU3				0.844									0.763
SCU1			0.827										0.791
SCU2			0.826										0.809
SCU3			0.750										0.746
PRO1								0.804					0.727
PRO2								0.844					0.767
PRO3								0.835					0.715
PLA1										0.845			0.751
PLA2										0.840			0.754
PLA3										0.797			0.701
PEO1					0.822								0.775
PEO2					0.810								0.760
PEO3					0.795								0.772
PER1									0.767				0.729
PER2									0.771				0.745
PER3									0.825				0.779
NAR1											0.763		0.686
NAR2											0.829		0.738
NAR3											0.796		0.745
CDE1							0.873						0.787
CDE2							0.813						0.705
CDE3							0.820						0.731
VEX1	0.856												0.765
VEX2	0.913												0.866
VEX3	0.879												0.809
SDE1												0.767	0.669
SDE2												0.837	0.738
SDE3												0.814	0.703
INT1		0.821											0.723
INT2		0.851											0.759
INT3		0.826											0.735

Extraction method: principal component analysis. Rotation method: Varimax with Kaiser normalization. Rotation converged in seven iterations.

After Varimax rotation with Kaiser normalization, the component structure also showed clear separation across the 12 dimensions, with each item loading most strongly on its theoretically corresponding component. Varimax was used to facilitate interpretation of the exploratory component solution; its orthogonal rotation was not taken as evidence that the underlying constructs were independent. Taken together, these results provide exploratory support for the construct structure of the scale and a reasonable basis for the subsequent confirmatory factor analysis (CFA) and SEM analysis.

#### 3.5.3 Confirmatory factor analysis (CFA)

##### 3.5.3.1 Measurement model fit evaluation

**C**FA was used to assess how well the 12-factor measurement model fit the data. The CFA was estimated using maximum likelihood (ML) estimation in AMOS. A normality assessment conducted during revision yielded a multivariate kurtosis value of 15.612 (critical ratio = 2.863), indicating a departure from multivariate normality. As shown in Table 10, the results indicated a good fit between the theoretical measurement model and the observed data: both χ^2^/df (1.104), and RMSEA (0.017) indicated good model fit. In addition, the fit indices CFI (0.990), TLI (0.988), IFI (0.990), GFI (0.922), AGFI (0.902) met commonly accepted criteria. Taken together, these results indicate that the proposed 12-factor measurement model (Figure 2) demonstrated satisfactory fit to the observed data, providing support for the subsequent validity assessment and structural model analysis.

**Table 10:** Measurement model fit indices.

Index	Recommended value	Model metrics	Conclusion
χ^2^/df	1 < χ^2^/df < 5	1.104	Acceptable
RMSEA	< 0.08	0.017	Acceptable
GFI	>0.85	0.922	Acceptable
CFI	>0.90	0.990	Acceptable
IFI	>0.90	0.990	Acceptable
TLI	>0.90	0.988	Acceptable
AGFI	>0.80	0.902	Acceptable

**Figure 2:**
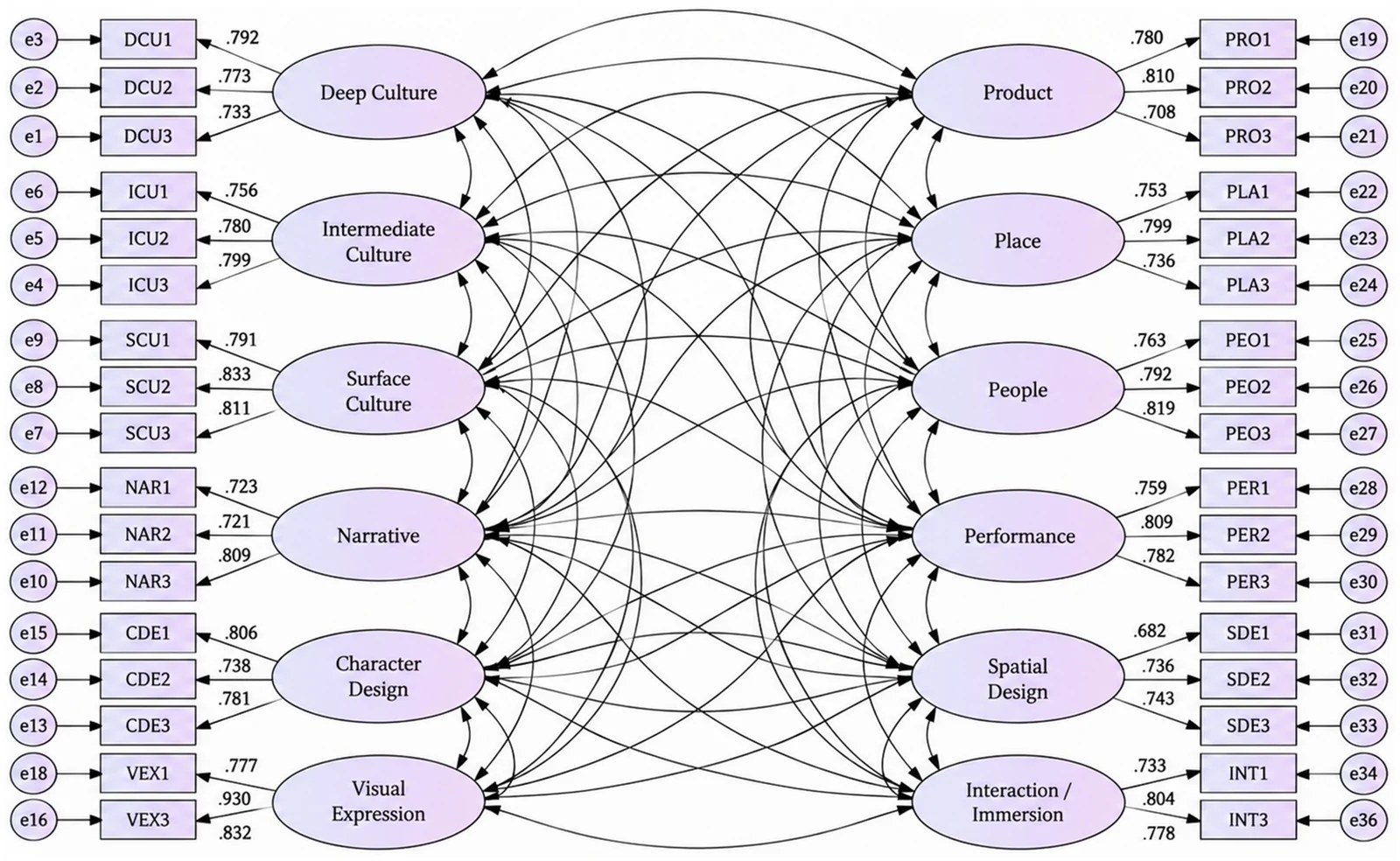
Confirmatory factor analysis (CFA) model with standardized estimates. The model illustrates the measurement structure between observed indicators and latent constructs and was used to assess reliability and validity based on responses from 368 industry professionals.

##### 3.5.3.2 Convergent validity and composite reliability

The composite reliability (CR) ranged from 0.7642 to 0.8848, and the average variance extracted (AVE) ranged from 0.5196 to 0.7203. The CR and AVE values met the commonly used minimum thresholds of 0.70 and 0.50, respectively, although their magnitudes varied across constructs (Bagozzi and Yi, 1988; Pallant, 2020 refer to Table 11). The results indicate acceptable internal consistency and convergent validity across the 12 constructs. However, Spatial Design showed comparatively modest measurement properties, with a CR of 0.7642 and an AVE of 0.5196. Its item SDE1 had a standardized loading of 0.682, slightly below the conventional benchmark of 0.70. In other words, the measurement model demonstrated adequate convergent validity and reliability, providing a sound basis for the subsequent discriminant validity assessment and structural model analysis.

**Table 11:** Confirmatory factor analysis and convergent validity metrics.

Dimension	Items	Standardized loading	S.E.	*Z*	*p*	AVE	CR
Deep Culture	DCU3	0.733	—	—	—	0.5874	0.8101
	DCU2	0.773	0.077	12.774	^***^		
	DCU1	0.792	0.079	12.913	^***^		
Intermediate Culture	ICU3	0.799	—	—	—	0.6061	0.8219
	ICU2	0.780	0.074	13.765	^***^		
	ICU1	0.756	0.072	13.500	^***^		
Surface Culture	SCU3	0.811	—	—	—	0.6591	0.8529
	SCU2	0.833	0.063	16.512	^***^		
	SCU1	0.791	0.063	15.758	^***^		
Product	PRO1	0.780	0.090	12.382	^***^	0.5886	0.8106
	PRO2	0.810	0.095	12.498	^***^		
	PRO3	0.708	—	—	—		
Place	PLA1	0.753	0.082	12.309	^***^	0.5824	0.8069
	PLA2	0.799	0.083	12.536	^***^		
	PLA3	0.736	—	—	—		
People	PEO1	0.768	0.066	14.688	^***^	0.6293	0.8358
	PEO2	0.792	0.063	15.073	^***^		
	PEO3	0.819	—	—	—		
Performance	PER1	0.759	0.067	14.005	^***^	0.6140	0.8267
	PER2	0.809	0.066	14.738	^***^		
	PER3	0.782	—	—	—		
Narrative	NAR1	0.723	0.073	12.563	^***^	0.5657	0.7957
	NAR2	0.721	0.069	12.535	^***^		
	NAR3	0.809	—	—	—		
Interaction/ Immersion	INT3	0.778	—	—	—	0.5963	0.8157
	INT2	0.804	0.078	13.407	^***^		
	INT1	0.733	0.075	12.818	^***^		
Character Design	CDE1	0.806	0.079	13.548	^***^	0.6014	0.8189
	CDE2	0.738	0.072	12.983	^***^		
	CDE3	0.781	—	—	—		
Visual Expression	VEX1	0.777	0.057	17.011	^***^	0.7203	0.8848
	VEX2	0.930	0.058	19.621	^***^		
	VEX3	0.832	—	—	—		
Spatial Design	SDE1	0.682	0.087	10.436	^***^	0.5196	0.7642
	SDE2	0.736	0.088	10.706	^***^		
	SDE3	0.743	—	—	—		

^***^p < 0.001.

##### 3.5.3.3 Discriminant validity analysis

With regard to Discriminant Validity, Table 12 presents the correlation matrix assessed according to the Fornell–Larcker criterion (Fornell and Larcker, 1981). The data showed that the square root of the AVE for each construct was greater than its correlations with the other constructs, supporting satisfactory discriminant validity among the 12 dimensions.

**Table 12:** Discriminant validity matrix (Fornell–Larcker criterion).

Construct	1	2	3	4	5	6	7	8	9	10	11	12
D	0.766											
I	0.091	0.779										
S	0.406	0.141	0.812									
Product	0.280	0.193	0.163	0.767								
Place	0.127	0.125	0.265	0.096	0.763							
People	0.357	0.381	0.361	0.275	0.161	0.793						
Performance	0.447	0.173	0.592	0.273	0.255	0.441	0.784					
Narrative	0.436	0.211	0.466	0.265	0.103	0.445	0.406	0.752				
Character Design	0.061	0.417	0.107	0.015	0.091	0.295	0.159	0.036	0.776			
Visual Expression	0.120	0.070	0.282	0.289	0.057	0.172	0.147	0.144	0.105	0.849		
Spatial Design	0.250	0.109	0.135	0.105	0.342	0.020	0.188	0.141	0.042	−0.055	0.721	
Interaction/ Immersion	0.116	−0.040	0.360	0.129	0.225	0.036	0.373	0.105	0.039	0.099	0.013	0.772

In response to concerns regarding potential conceptual overlap among theoretically adjacent constructs, discriminant validity was further assessed using the heterotrait–monotrait ratio of correlations (HTMT). All HTMT values were below the conservative threshold of 0.85 (Henseler et al., 2015), with the maximum value being 0.590 between Surface Culture and Performance (Table S1). Of particular relevance, the HTMT values were 0.436 between Deep Culture and Narrative and 0.285 between Surface Culture and Visual Expression. These results provide additional evidence that the constructs are empirically distinguishable despite their theoretical relatedness.

Taken together, these results support the 12 dimensions (3 Cultural levels, 4P Elements, 5 Animation Design dimensions) at the measurement level, providing an appropriate basis for the subsequent model path tests.

#### 3.5.4 Common-method bias assessment

To assess the potential influence of common method variance, Harman's single-factor test was conducted using an unrotated principal component analysis in which a single component was extracted (Podsakoff et al., 2003). The single component had an eigenvalue of 6.754 and accounted for 18.762% of the total variance. Because this proportion was substantially below the commonly used 50% benchmark, the result did not indicate severe single-factor dominance. However, Harman's single-factor test provides only a limited diagnostic and cannot rule out common method bias. Because the study relied on questionnaire responses collected from the same respondents at a single time point, the possibility of common method bias remains a limitation when interpreting the observed relationships.

## 4 Structural equation modeling analysis and hypotheses testing

### 4.1 Structural model evaluation

After establishing the reliability and validity of the measurement model, this study used AMOS 26.0 to construct the structural model. The structural equation model (SEM) was estimated using maximum likelihood (ML) estimation; its normality assessment yielded the same multivariate kurtosis and critical ratio reported for the CFA in Section 3.5.3.1. The model was then used to test the hypothesized relationships in the theoretical framework, particularly the relationships between cultural factors, including cultural layers and the 4P taxonomy, and animation design outcomes. The full structural model and hypothesized paths are presented in Figure 3.

**Figure 3:**
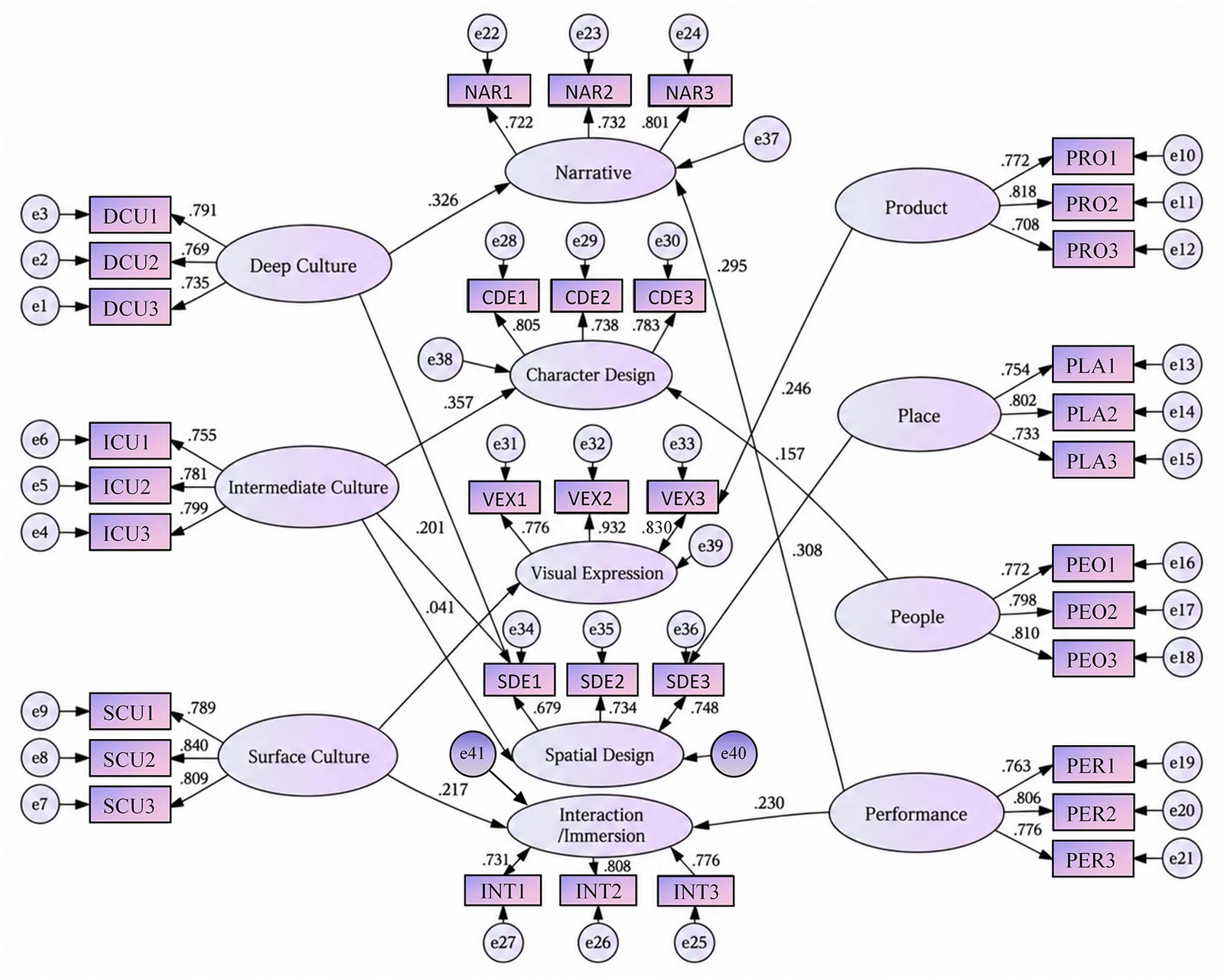
Structural model with standardized path coefficients. Values on the structural paths are standardized path coefficients (β). The Intermediate Culture → Spatial Design path was not statistically significant.

A battery of standard absolute, incremental, and parsimonious fit indices (Table 13) corroborated the overall fit test of the data-model before interpreting the specific structural coefficients. As summarized in Table 13, the model demonstrated satisfactory overall fit. The chi-square to degrees-of-freedom ratio (χ^2^/df) was 1.165 and the RMSEA (Root Mean Square Error of Approximation) was 0.021. In addition, the GFI (0.913), AGFI (0.897), CFI (0.983), IFI (0.983), and NFI (0.892), met the recommended acceptance thresholds. The parsimonious fit indices, including PNFI (0.796) and PCFI (0.877), also indicated satisfactory model fit. Therefore, these results support the adequacy of the structural model and provide an appropriate basis for subsequent hypothesis testing.

**Table 13:** Structural model fit indices.

Category	Index	Recommended value	Model metrics	Conclusion
Absolute fit indices	χ^2^/df	1 < χ^2^/df < 5	1.165	Acceptable
	RMSEA	< 0.08	0.021	Acceptable
	GFI	>0.85	0.913	Acceptable
	AGFI	>0.80	0.897	Acceptable
Incremental fit indices	CFI	>0.90	0.983	Acceptable
	IFI	>0.90	0.983	Acceptable
	NFI	>0.80	0.892	Acceptable
Parsimonious fit indices	PNFI	>0.50	0.796	Acceptable
	PCFI	>0.50	0.877	Acceptable

Table 14 presents descriptive statistics for the 12 constructs and collinearity diagnostics based on their composite scores. Each construct score was calculated as the mean of its three corresponding items, and VIFs were obtained from separate linear regressions using the predictors specified for each endogenous construct in the structural model; the VIF values ranged from 1.014 to 1.323, indicating no substantial multicollinearity among these predictors.

**Table 14:** Construct descriptive statistics and collinearity diagnostics.

Construct	*N*	*M*	SD	Endogenous construct	VIF
Deep Culture	368	3.0879	1.14963	Narrative	1.153
Deep Culture	368	—	—	Spatial Design	1.015
Intermediate Culture	368	3.0670	1.15275	Character Design	1.109
Intermediate Culture	368	—	—	Spatial Design	1.014
Surface Culture	368	3.3424	1.27004	Visual Expression	1.018
Surface Culture	368	—	—	Interaction/Immersion	1.323
Product	368	2.9076	1.03053	Visual Expression	1.018
Place	368	3.1395	1.03285	Spatial Design	1.019
People	368	3.1368	1.26374	Character Design	1.109
Performance	368	2.9194	1.27856	Narrative	1.153
Performance	368	—	—	Interaction/Immersion	1.323
Narrative	368	3.0851	1.09079	—	—
Character Design	368	3.0226	1.01388	—	—
Visual Expression	368	3.0417	1.08944	—	—
Spatial Design	368	3.0489	0.93216	—	—
Interaction/Immersion	368	3.0725	1.02830	—	—

*M*, mean; SD, standard deviation; VIF, variance inflation factor.

Construct scores were calculated as the mean of their three corresponding questionnaire items. VIFs were obtained from separate linear regressions using the construct scores of predictors specified for each endogenous construct in the structural model. A dash indicates that the statistic is already reported in an earlier row for the same construct or is not applicable.

### 4.2 Hypothesis testing and path analysis

Table 15 reports the AMOS 26.0 estimates of the structural paths used to test the hypothesized relationships. The reported results include unstandardized path coefficients (B), standardized path coefficients (β), standard errors (S.E.), critical ratios (C.R.), *p*-values, and bias-corrected 95% bootstrap confidence intervals. Bias-corrected 95% bootstrap confidence intervals based on 2,000 bootstrap samples were examined to provide uncertainty estimates for the structural path coefficients.

**Table 15:** Structural model path coefficients and hypothesis testing results.

Path	*B*	β	S.E.	C.R.	*P*	95% BC CI for *B*	Hypothesis	Result
Deep Culture → Narrative	0.303	0.326	0.067	4.536	^***^	[0.165, 0.467]	H1a	Supported
Deep Culture → Spatial Design	0.150	0.201	0.049	3.074	0.002	[0.049, 0.261]	H1b	Supported
Intermediate Culture → Character Design	0.333	0.357	0.064	5.185	^***^	[0.206, 0.462]	H2a	Supported
Intermediate Culture → Spatial Design	0.030	0.041	0.047	0.652	0.514	[−0.061, 0.127]	H2b	Not supported
Surface Culture → Visual Expression	0.193	0.233	0.048	3.989	^***^	[0.098, 0.298]	H3a	Supported
Surface Culture → Interaction/immersion	0.168	0.217	0.063	2.692	0.007	[0.044, 0.289]	H3b	Supported
Product → Visual Expression	0.279	0.246	0.069	4.045	^***^	[0.135, 0.429]	H4a	Supported
Place → Spatial Design	0.263	0.308	0.059	4.474	^***^	[0.132, 0.417]	H4b	Supported
People → Character Design	0.131	0.157	0.055	2.398	0.016	[0.018, 0.248]	H4c	Supported
Performance → Narrative	0.239	0.295	0.057	4.223	^***^	[0.110, 0.368]	H4d	Supported
Performance → Interaction/Immersion	0.173	0.230	0.062	2.810	0.005	[0.054, 0.309]	H4e	Supported

^***^p < 0.001.

#### 4.2.1 Relationships between cultural layers and animation design elements

**T**he structural model generally supported the role of cultural layers in animation production. The results showed that Deep Culture had significant positive associations with Narrative (β = 0.326, *p* < 0.001) and Spatial Design (β = 0.201, *p* = 0.002), supporting H1a and H1b. Intermediate Culture was significantly associated with Character Design (β = 0.357, *p* < 0.001), supporting H2a; however, its association with Spatial Design was not statistically significant (β = 0.041, *p* = 0.514) and thus H2b was not supported. Finally, the Surface Culture was significantly and positively associated with Visual Expression (β = 0.233, *p* < 0.001) and Interaction/Immersion (β = 0.217, *p* = 0.007), supporting H3a and H3b.

#### 4.2.2 Relationships between 4P cultural content and animation design elements

The content-oriented 4P taxonomy complements the cultural layers by organizing cultural content into Product, Place, People, and Performance. Path coefficients showed differentiated associations between the 4P dimensions and animation design dimensions: Product attributes were significantly and positively associated with Visual Expression (β = 0.246, *p* < 0.001, supporting H4a); Place was significantly and positively associated with Spatial Design (β = 0.308, *p* < 0.001, supporting H4b); and People were positively related to Character Design (β = 0.157, *p* = 0.016, supporting H4c). Finally, Performance was significantly and positively associated with two dimensions: Narrative (β = 0.295, *p* < 0.001) and Interaction/Immersion (β = 0.230, *p* = 0.005). These results provide support for H4d and H4e. The confidence intervals for the ten statistically supported paths did not include zero, whereas the interval for the Intermediate Culture → Spatial Design path included zero, consistent with the non-significant maximum-likelihood estimate. Together, the empirical findings support ten of the eleven hypothesized pathways within the dual-cultural framework. The structural model accounted for 27.8% of the variance in Narrative, 19.4% in Character Design, 13.3% in Visual Expression, 15.8% in Spatial Design, and 16.0% in Interaction/Immersion.

## 5 Discussion and conclusion

### 5.1 Discussion of core findings

A primary theoretical contribution of this manuscript is that it combines a layered conception of culture with a content-oriented 4P taxonomy to form a unified framework for animation design. In addition to providing the empirical support for most of the proposed structural pathways, the empirical evidence sheds light on the specific relationships between cultural layers and dimensions of digital media design. Finally, these results contribute to a quantitative bridge between abstract cultural concepts and concrete design practice.

#### 5.1.1 The mapping of cultural layers to animation design

The results suggest differentiated relationships between cultural levels and the design dimensions. Deep Culture was significantly associated with Narrative development and Spatial Design. Regarding its association with Narrative (β = 0.326), the findings suggest that culturally grounded animation narratives do not derive only from the external cultural manifestations; rather, they are closely related to underlying philosophical perspectives, beliefs, and basic social values. The relationship between Deep Culture and Spatial Design (β = 0.201) further suggests that underlying cultural conceptions of spatial and cosmic order may be related to the organization and meaning of virtual environments.

As for the Intermediate Culture dimension, it was significantly and positively associated with Character Design (β = 0.357, *p* < 0.001), suggesting that behavioral norms, moral paradigms, and patterns of social interaction may be related to character construction. Animated characters may therefore derive part of their social and cultural credibility not merely from superficial cultural features, but also from culturally embedded customs and social interactions. However, the hypothesized association of Intermediate Culture with Spatial Design was not statistically significant (β = 0.041, *p* = 0.514), and H2b was therefore not supported.

Surface Culture, in contrast, was significantly associated with Visual Expression (β = 0.233) and Interaction/Immersion (β = 0.217). Observable cultural elements, such as color scheme, traditional clothing, and architectural motifs, may therefore serve as accessible design resources for communicating cultural context through visual and interaction/immersion-related design.

#### 5.1.2 The 4P as a practical toolkit for cultural translation

The 4P elements offer a structured way to relate abstract cultural concepts to tangible design applications. The results showed clear associations between the 4P elements and specific design dimensions. “Product” (P1) was significantly and positively associated with Visual Expression (β = 0.246, *p* < 0.001), while “Place” (P2) was significantly and positively associated with Spatial Design (β = 0.308, *p* < 0.001). These findings suggest that cultural translation in animation can be supported through both material objects and geographical contexts, providing concrete resources for visual and environmental design.

One finding that stands out concerns the relationships associated with Character Design. Although “People” (P3) was significantly and positively associated with Character design (β = 0.157), Character Design was also significantly associated with Intermediate Culture (β = 0.357). Taken together, these findings suggest that culturally grounded Character Design may reflect not only demographic or externally observable characteristics, but also social norms, moral logic, and patterns of social interaction. For animation designers, incorporating these intermediate cultural dimensions may help create more culturally grounded characters while reducing reliance on superficial cultural stereotyping.

“Performance” (P4) also showed significant positive associations with both Narrative (β = 0.295) and Interaction/Immersion (β = 0.230). These findings suggest that dynamic cultural practices, such as rituals and festivals, may provide useful resources for narrative development and design features related to interaction/immersion. In this sense, Performance may provide a practical dimension through which dynamic cultural practices can be represented in animation design.

### 5.2 Real-world application of structural relationships

Although we have identified relationships between culture and design through structural analysis, an illustrative case can further demonstrate how these relationships may operate in context. *I Am What I Am* (2021) was selected as an illustrative example because its representations of Chinese traditional culture, including cultural settings and practices, are closely related to the focus of this study. The interpretation is based on visual observation and description of the scenes shown in Figures 4, 5, with the observed elements discussed in relation to the relevant framework dimensions; no separate coding procedure was undertaken. Using *I Am What I Am* (2021) as an example, we can see that the Place and Product are also vividly portrayed as particular kinds of visual spectacle. As in Figure 4, layers of clan flags and drum formations are all material manifestations of culture and aesthetics; they are not merely props to set an atmosphere; but also make visible the deeper social relationship of “clan belief and confrontation” rooted in the local cultural context. The dotted connection between Intermediate Culture and Spatial Design in Figure 4 represents a case-specific observation rather than a statistically supported structural relationship. The emotional center of this sequence is the digital rendering of the lion dance. These elements ground the cultural context in a concrete spatial anchor within the sequence, readying the scene for the impending ritual. The SEM results showed a significant positive relationship between Performance and Interaction/Immersion (β = 0.230, *p* = 0.005). In design practice, this is represented through the embodied semiotics of cooperation. The success of the “high-pole leap” lies in the intentionality and physical synchronization between the “head” and “tail” performers (Figure 5).

**Figure 4:**
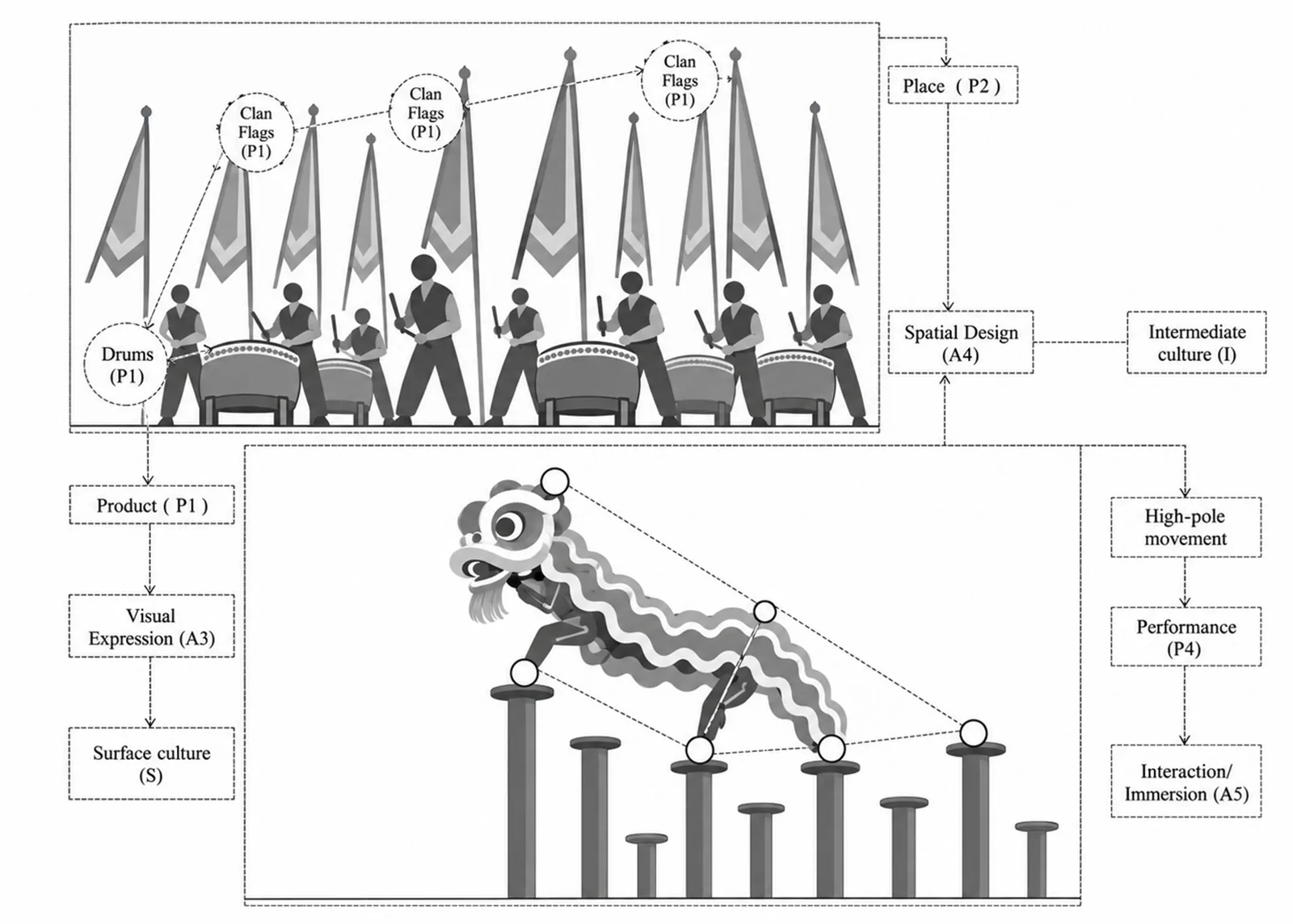
Author-created schematic illustration developed to conceptually illustrate the authors' analysis of *I Am What I Am* (2021). This figure is an original schematic drawing prepared by the authors. It illustrates how selected cultural and design dimensions can be interpreted within the proposed dual-cultural framework. The dotted connection between Intermediate Culture and Spatial Design represents a contextual observation in this case rather than a statistically supported structural relationship.

**Figure 5:**
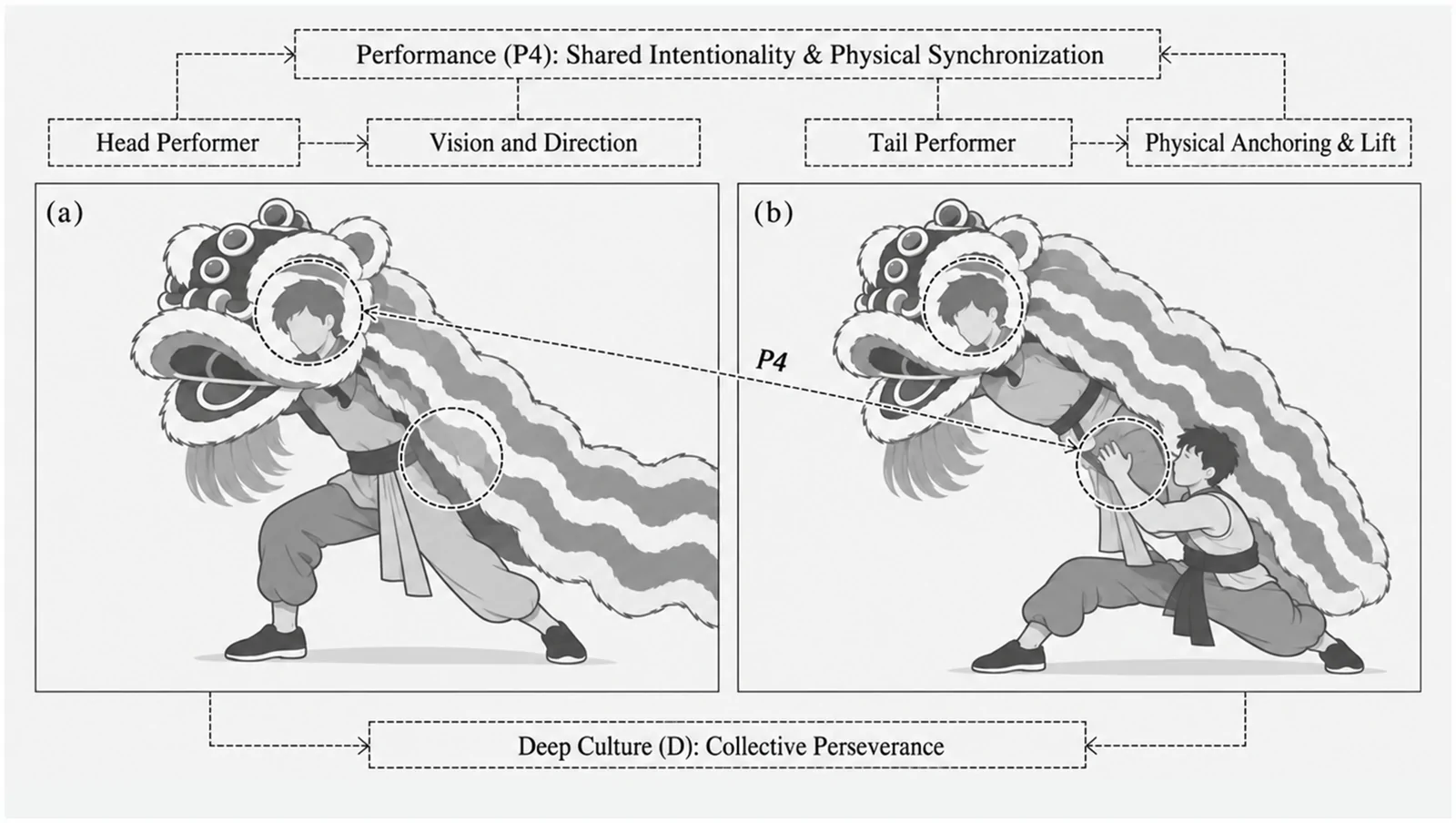
Author-created schematic illustration developed to conceptually illustrate the authors' analysis of embodied coordination in *I Am What I Am* (2021). This figure is an original schematic drawing prepared by the authors. It illustrates how mental direction is aligned with physical movement through the coordinated actions of two performers: **(a)** the head performer, who guides vision and direction, and **(b)** the tail performer, who provides physical anchoring and lift. The figure highlights shared intentionality, bodily synchronization, and the cultural value of collective perseverance.

By rendering the visceral realities of the ritual, including the restricted vision within the bamboo frame of the lion head and the upward lift executed by the tail performer, the animation makes use of the Performance dimension to aid cultural translation as a physical expression of deeper cultural values including collective perseverance and sacrifice. This case illustration provides a contextual interpretation of the relationship between Performance and Interaction/Immersion observed in the structural model. Rather than functioning only as visual spectacle, the sequence can be interpreted as representing the collective struggle and solidarity embodied in the performance.

### 5.3 Theoretical contributions

This study makes three main theoretical contributions at the intersection of digital culture and animation design. First, it develops an interdisciplinary dual-cultural framework by bringing together a layered conception of culture and a content-oriented 4P taxonomy. In this paper, we combine these two complementary analytical perspectives, moving beyond a one-dimensional focus on cultural representation toward consideration of both cultural meanings and their design manifestations. The dual-cultural framework therefore helps bridge the gap between abstract cultural concepts and systematic design practice.

Building on this framework, the research identifies differentiated relationships between cultural depth and design dimensions. The results suggest that different levels of culture (Deep, Intermediate, and Surface) are associated with particular dimensions of the digital design, although not all hypothesized relationships were supported. This provides a structured perspective for examining how different levels of cultural meaning may be reflected in digital media design.

Beyond these relationships, this study provides a structured perspective on how intangible cultural meanings may be related to tangible design dimensions. The structural analysis shows differentiated associations between the 4P elements and animation design dimensions. In particular, Performance represents dynamic cultural practices and was significantly associated with both Narrative and Interaction/Immersion, illustrating how embodied cultural activities may be related to narrative and interaction/immersion-related dimensions of animation design.

### 5.4 Practical implications for applied animation design

This paper uses the dual-cultural framework to provide a structured reference for applying cultural concepts to animation design, as illustrated by the animation cases discussed above. First, at the initial stage of idea development, a team can consider a value-oriented design logic. Rather than relying on a superficial accumulation of visual symbols, the animation's structural foundation—namely, its narrative logic and spatial rules—can be considered in relation to Deep Culture, such as core philosophical beliefs and worldviews.

The second point concerns the Character Design and Visual Expression stage. The framework suggests that creators can consider character design beyond physical appearance by incorporating internal traits and social-cultural characteristics associated with Intermediate Culture. Visual expression can incorporate culturally recognizable elements related to the 4P taxonomy, such as traditional clothing and festive colors, as well as other Surface Culture elements. Such an approach may help creators integrate character-related and visual cultural elements more systematically.

On a broader project management level, the dual-cultural framework may also serve as an analytical reference during project development. During IP incubation and early review phases, studios may use it to map relevant cultural and design dimensions within a project. Identifying potential conceptual gaps before entering production may help teams consider cultural meanings more systematically throughout the design process.

### 5.5 Limitations and future research

Although the findings provide preliminary empirical support for the dual-cultural framework, there are theoretical and methodological boundaries to be explored in future research. One is that the study used cross-sectional data; thus, the model results cannot be regarded as definitive evidence of causal relationships. In the future, longitudinal studies may be adopted to examine how these relationships change over time. The second limitation is that the respondents were drawn from animation- or culture-related professional fields, which may limit the generalizability of the findings. Further study could include data from general audiences in order to compare the perspective of the design end and receiving end. Third, the present study mainly examines direct paths. Future research could add audience psychological characteristics, such as emotions, cognition, and cultural identification, into the model as a mediator or moderator variable, to examine psychological processes not directly measured in the present study. Finally, although the questionnaire provided culturally themed animation examples as a common reference context, respondents could also draw on other culturally themed animated works familiar to them. Therefore, the referenced works were not fully standardized across participants. Future studies could use standardized animation stimuli in experimental evaluations of audience responses and test measurement invariance across cultural groups before comparing their construct scores or structural relationships. In addition, the exploratory PCA assessment, CFA, and SEM were conducted using the same sample; therefore, future research should examine the measurement and structural models using an independent sample to provide external validation.

A further measurement-related limitation is that some cultural and animation-design indicators use conceptually related wording, which may contribute to measurement overlap despite the acceptable discriminant-validity results. Future scale development should further differentiate cultural-content indicators from animation-design indicators and validate the revised measures using an independent sample. The single-source questionnaire design may also be susceptible to common-method variance; although Harman's single-factor assessment did not indicate severe single-factor dominance, future studies should adopt stronger procedural or statistical controls for common-method bias. The present study did not compare the proposed structural model with alternative model specifications; therefore, the reported fit and path estimates should not be interpreted as establishing that the proposed specification is uniquely supported by the data. Future studies could also compare the proposed specification with fuller or competing structural models, including theoretically plausible cross-domain paths, using independent samples.

## 6 Conclusion

In this study, we proposed and examined a dual-cultural analytical framework in order to connect relatively abstract cultural concepts with the practical realities of digital design. This article combines a layered conception of culture with a content-oriented 4P taxonomy as the theoretical framework for examining their relationships with animation design. Deep and Intermediate Culture represent relatively deeper cultural meanings that may be associated with narrative, spatial, and character-related dimensions of animation design. Surface Culture, together with the Product, Place, People, and Performance dimensions of the 4P taxonomy, represents more observable forms through which cultural content may be expressed in animation design. Rather than relying on isolated, uni-dimensional metrics, this study adopts a structurally integrated perspective. It brings these complementary analytical perspectives together in the study of culturally informed animation design and provides preliminary empirical evidence, based on self-report ratings from professionals in animation- or culture-related fields, regarding the relationships between cultural and animation design dimensions. These findings should be interpreted as preliminary and in light of the measurement and reporting concerns identified in this study. For animation practitioners, the framework may provide a structured reference for considering cultural meanings beyond surface-level symbols and relating them to different dimensions of animation design.

## Data Availability

The original contributions presented in the study are included in the article/Supplementary material, further inquiries can be directed to the corresponding author.
