# Supplementary material for: Translating culture into design practice: a preliminary test of a dual-cultural framework for animation
Source: Front Psychol. 2026 Oct 20;17:1882608. doi: 10.3389/fpsyg.2026.1882608 (PMC13731867; doi:10.3389/fpsyg.2026.1882608)
Supplement: Supplementary file 1 [file Supplementary_file_1.docx]

# Supplementary Materials

## Appendix S1

## Measurement Construct Mapping Table

This appendix presents the mapping between theoretical constructs, latent variables used in the structural equation model (SEM), and their corresponding observed measurement items. All variable codes are fully consistent with the dataset and SEM estimation results.

| Construct Category | Theoretical Construct | Latent Variable Code | Observed Items |
| --- | --- | --- | --- |
| Cultural Layers | Deep Culture | D | DCU1, DCU2, DCU3 |
|  | Intermediate Culture | I | ICU1, ICU2, ICU3 |
|  | Surface Culture | S | SCU1, SCU2, SCU3 |
| 4P Cultural Content | Product | P1 | PRO1, PRO2, PRO3 |
|  | Place | P2 | PLA1, PLA2, PLA3 |
|  | People | P3 | PEO1, PEO2, PEO3 |
|  | Performance | P4 | PER1, PER2, PER3 |
| Animation Design Elements | Narrative | A1 | NAR1, NAR2, NAR3 |
|  | Character Design | A2 | CDE1, CDE2, CDE3 |
|  | Visual Expression | A3 | VEX1, VEX2, VEX3 |
|  | Spatial Design | A4 | SDE1, SDE2, SDE3 |
|  | Interaction/Immersion | A5 | INT1, INT2, INT3 |

All observed variables were measured using a five-point Likert scale (1 = strongly disagree, 5 = strongly agree). Each construct was operationalized using three measurement items, consistent with the SEM framework presented in the main text.

## Appendix S2. Survey Instrument

This questionnaire was designed to examine the associations between cultural dimensions and animation design elements (e.g., Narrative, Character Design, Visual Expression , Spatial Design, and Interaction/Immersion).

Participants were asked to evaluate a culturally distinctive animated work based on their viewing experience. **The original questionnaire provided several culturally themed animated works as reference examples, including Big Fish & Begonia, Spirited Awa**y**, and Ne Zha (**Ne Zha: Birth of the Demon Child**), while participants could base their responses on other culturally themed animated works familiar to them.** Given that the participants had professional backgrounds in animation, design, cultural studies, or related fields, the questionnaire adopted discipline-specific terms to capture nuanced evaluations of cultural representation and animation design.

All items were measured using a five-point Likert scale:

1 = Strongly Disagree; 2 = Disagree; 3 = Neutral; 4 = Agree; 5 = Strongly Agree.

## Section A. Cultural Layers

### Deep Culture (DCU)

1. The animation appears to reflect a coherent philosophical or belief system.

2. The story theme embodies specific cultural values.

3. The characters’ behaviors reflect moral judgments embedded in the culture.

### Intermediate Culture (ICU)

1. Interactions between characters conform to culturally specific etiquette.

2. The spatial arrangement reflects the daily life logic of the culture.

3. The narrative rhythm or behavioral patterns correspond to real cultural traditions.

### Surface Culture (SCU)

1. Costumes, totems, or language exhibit distinctive regional characteristics.

2. The use of colors and symbols reflects strong cultural stylistic features.

3. Architectural and decorative designs clearly communicate cultural identity.

## Section B. 4P Cultural Content

### Product (P1)

1. Cultural artifacts or culturally related objects are displayed in the animation.

2. Certain objects in the story carry symbolic cultural meanings.

3. The design of everyday objects reflects traditional cultural characteristics.

### Place (P2)

1. The background scenes exhibit clear regional cultural identity.

2. The spatial layout reflects the integration of local natural and cultural elements

3. The scenes evoke a specific cultural environment.

### People (P3)

1. The animated characters represent typical figures of a cultural group.

2. The characters’ language and behavioral habits align with local cultural practices.

3. Social roles appear to correspond to specific cultural frameworks.

### Performance (P4)

1. The animation incorporates cultural behaviors such as festivals or rituals.

2. Music or dance styles are culturally representative.

3. The narrative rhythm reflects culturally specific modes of expression.

## Section C. Animation Design Components

### Narrative (A1)

1. The story structure is influenced by cultural myths or legends.

2. The narrative presents culturally meaningful oppositions or transformations.

3. Cultural values play a key role in plot progression.

### Character Design (A2)

1. Character design conveys distinct cultural identity.

2. Appearance and costume design correspond to cultural traditions.

3. Character background and behavioral logic align with the cultural context.

### Visual Expression (A3)

1. The overall visual style reflects cultural aesthetics.

2. Patterns and decorations incorporate traditional local elements.

3. Color selection and lighting rhythm have symbolic cultural meaning.

### Spatial Design(A4)

1. Scene construction reflects cultural spatial symbolism.

2. Architecture and natural landscapes integrate traditional concepts.

3. Spatial hierarchy and movement pathways reflect local structural logic.

### Interaction/Immersion (A5)

1. Music or sound details enhance the cultural atmosphere.

2. Visual or rhythmic elements make the audience feel involved in cultural rituals.

3. The animation rhythm creates a sense of immersion in cultural festivities.

## Section D. Demographic Information (Optional)

### Demographics

1. Gender: Male / Female / Other

2. Age Group: 18–24 / 25–34 / 35–44 / 45+

3. Working in animation or culture-related field: Yes / No

4. Most impressive culturally distinctive animated work: ____________
