# Supplementary material for: Translating culture into design practice: a preliminary test of a dual-cultural framework for animation
Source: Front Psychol. 2026 Oct 20;17:1882608. doi: 10.3389/fpsyg.2026.1882608 (PMC13731867; doi:10.3389/fpsyg.2026.1882608)
Supplement: Supplementary file 2 [file Table_1.docx]

**Supplementary Table S1**

Table S1. HTMT Discriminant Validity Assessment

| **Construct** | **D** | **I** | **S** | **PRO** | **PLA** | **PEO** | **PER** | **NAR** | **CDE** | **VEX** | **SDE** | **INT** |
| --- | --- | --- | --- | --- | --- | --- | --- | --- | --- | --- | --- | --- |
| **D** | — |  |  |  |  |  |  |  |  |  |  |  |
| **I** | .116 | — |  |  |  |  |  |  |  |  |  |  |
| **S** | .408 | .141 | — |  |  |  |  |  |  |  |  |  |
| **PRO** | .275 | .187 | .160 | — |  |  |  |  |  |  |  |  |
| **PLA** | .126 | .123 | .266 | .106 | — |  |  |  |  |  |  |  |
| **PEO** | .372 | .379 | .358 | .265 | .169 | — |  |  |  |  |  |  |
| **PER** | .446 | .175 | **.590** | .268 | .258 | .444 | — |  |  |  |  |  |
| **NAR** | **.436** | .215 | .476 | .249 | .099 | .436 | .412 | — |  |  |  |  |
| **CDE** | .071 | **.422** | .110 | .056 | .096 | .297 | .164 | .053 | — |  |  |  |
| **VEX** | .123 | .096 | **.285** | .295 | .075 | .185 | .149 | .140 | .115 | — |  |  |
| **SDE** | .254 | .106 | .133 | .103 | **.350** | .049 | .193 | .137 | .063 | .066 | — |  |
| **INT** | .124 | .050 | .361 | .137 | .227 | .050 | **.371** | .128 | .045 | .093 | .117 | — |

**Note:** HTMT = heterotrait–monotrait ratio of correlations. All values are below the conservative threshold of .85.
